# Differences Between MR Brain Region Segmentation Methods: Impact on Single-Subject Analysis

**DOI:** 10.3389/fdata.2021.577164

**Published:** 2021-07-30

**Authors:** W. Huizinga, D. H. J. Poot, E. J. Vinke, F. Wenzel, E. E. Bron, N. Toussaint, C. Ledig, H. Vrooman, M. A. Ikram, W. J. Niessen, M. W. Vernooij, S. Klein

**Affiliations:** ^1^ Biomedical Imaging Group Rotterdam, Department of Radiology & Nuclear Medicine and Medical Informatics, Erasmus MC, Rotterdam, Netherlands; ^2^ Department of Radiology & Nuclear Medicine, Erasmus MC, Rotterdam, Netherlands; ^3^ Department of Epidemiology, Erasmus MC, Rotterdam, Netherlands; ^4^ Philips Research Hamburg, Hamburg, Germany; ^5^ School of Biomedical Engineering, King's College London, London, United Kingdom; ^6^ Biomedical Image Analysis Group, Department of Computing, Imperial College London, London, United Kingdom; ^7^ Quantitative Imaging Group, Department of Imaging Physics, Faculty of Applied Sciences, Delft University of Technology, Delft, Netherlands

**Keywords:** brain region segmentation, subcortical, comparison study, normative modeling, magnetic resonance imaging

## Abstract

For the segmentation of magnetic resonance brain images into anatomical regions, numerous fully automated methods have been proposed and compared to reference segmentations obtained manually. However, systematic differences might exist between the resulting segmentations, depending on the segmentation method and underlying brain atlas. This potentially results in sensitivity differences to disease and can further complicate the comparison of individual patients to normative data. In this study, we aim to answer two research questions: 1) to what extent are methods interchangeable, as long as the same method is being used for computing normative volume distributions and patient-specific volumes? and 2) can different methods be used for computing normative volume distributions and assessing patient-specific volumes? To answer these questions, we compared volumes of six brain regions calculated by five state-of-the-art segmentation methods: Erasmus MC (EMC), FreeSurfer (FS), geodesic information flows (GIF), multi-atlas label propagation with expectation–maximization (MALP-EM), and model-based brain segmentation (MBS). We applied the methods on 988 non-demented (ND) subjects and computed the correlation (PCC-v) and absolute agreement (ICC-v) on the volumes. For most regions, the PCC-v was good ( >0.75 ), indicating that volume differences between methods in ND subjects are mainly due to systematic differences. The ICC-v was generally lower, especially for the smaller regions, indicating that it is essential that the same method is used to generate normative and patient data. To evaluate the impact on single-subject analysis, we also applied the methods to 42 patients with Alzheimer’s disease (AD). In the case where the normative distributions and the patient-specific volumes were calculated by the same method, the patient’s distance to the normative distribution was assessed with the z-score. We determined the diagnostic value of this z-score, which showed to be consistent across methods. The absolute agreement on the AD patients’ z-scores was high for regions of thalamus and putamen. This is encouraging as it indicates that the studied methods are interchangeable for these regions. For regions such as the hippocampus, amygdala, caudate nucleus and accumbens, and globus pallidus, not all method combinations showed a high ICC-z. Whether two methods are indeed interchangeable should be confirmed for the specific application and dataset of interest.

## 1 Introduction

Quantitative imaging biomarkers are biological features that can be measured using medical images. They are of interest for diagnosis when changes in these features are due to disease. In the case of traumatic brain injury or neurodegenerative disease, typical valuable quantitative imaging biomarkers are brain region volumes (Zagorchev et al., 2015; Ledig et al., 2015; Scheltens et al., 2002). A well-known example is the volume of the hippocampus. A relatively low volume may indicate the presence of Alzheimer’s disease (AD)’ (Convit et al., 1997; Jack et al., 1999; den Heijer et al., 2006). To determine if a patient deviates significantly, one can compare it to the so-called normative data (Brewer, 2009; Ziegler et al., 2014; Marquand et al., 2016). Normative data are acquired in a reference population, and they are used as baseline distribution for a measurement, against which an individual measurement can be compared. Normative data may incorporate covariates such as age or gender, when the distribution is expected to vary significantly as a function of these variables. Well-known examples are head-circumference-for-age, height-for-age, weight-for-age, and weight-for-height norms, provided by the WHO (de Onis et al., 2006), for detecting abnormal growth in children. The dependency on age is also the case for volumetric magnetic resonance (MR) brain images. Brewer (2009) proposed using quantile curves as a function of age as normative data for volumetric MR measurements.

Volumetric MR measurements are acquired by segmenting the brain into its different tissue types and regions of interest. The manual segmentation of a brain image is a time-consuming task, which has to be performed by an expert and is therefore too expensive and impractical for a clinical setting (Brewer (2009)). To automatically obtain brain region volumes from MRI brain data, numerous fully automated brain segmentation methods have been proposed in the literature. Each method relies on different techniques to segment either the full brain or a specific region. We can subdivide the methods that are based on prior probability maps (Fischl et al., 2002), statistical shape and appearance models (Babalola et al., 2008a; Patenaude et al., 2011; Wenzel et al., 2018), multi-atlas registration and labeling (Bron et al., 2014; Cardoso et al., 2015; Ledig et al., 2015; Murphy et al., 2014; Wang et al., 2014; Wolz et al., 2010; van der Lijn et al., 2008), deep learning approaches (Bao and Chung, 2018; Shakeri et al., 2016; de Brébisson and Montana, 2015), and other (Hammers et al., 2009; Corso et al., 2007; Morra et al., 2008; Tue et al., 2008). Each method aims to segment the brain as accurately as possible where manual segmentation serves as the gold standard. Various comparison studies have been performed with regard to automated brain segmentation methods. Grimm et al. (2015) assessed the differences in amygdalar and hippocampal volume resulting from Freesurfer (Fischl et al., 2002), VBM8 (VBM^1^), and manual segmentation. They concluded that volumes computed with VBM8 and Freesurfer V5.0 were comparable, and systematic and proportional differences were mainly due to different definitions of anatomic boundaries. They concluded that large differences can still exist even with high correlation coefficients. Morey et al. (2009) also compared amygdalar and hippocampal volumes but using methods such as FSL/FIRST 4.0.1^2^, Freesurfer 4.0.5 (Fischl et al., 2002), and manual segmentation. They concluded that for the hippocampus, Freesurfer was more similar to manual segmentation in terms of volume difference, overlap, and correlation. For the amygdala, FIRST represented the shape more accurately than Freesurfer. Babalola et al. (2008b) compared four different state-of-the-art algorithms for automatic segmentation of subcortical structures in MR brain images and evaluated spatial overlap, distance, and volumetric measures: classifier fusion and labeling (Aljabar et al., 2007), profile active appearance models (Babalola et al., 2007), Bayesian appearance models (Patenaude et al., 2011), and expectation–maximization–based segmentation using a dynamic brain atlas (Murgasova et al., 2006). They concluded that all four methods perform on par with recently published methods. One of their evaluating methods (Aljabar et al., 2007) performed significantly better than the other three methods according to their evaluation. Perlaki et al. (2017) compared the segmentation accuracy of the caudate nucleus and putamen between FSL/FIRST (version FSL’s build: 507) and Freesurfer (versions 4.5 and 5.3) by studying the Dice coefficient, and absolute and relative volume difference. They also measured consistency and absolute agreement. They concluded that for caudate segmentation, FIRST and Freesurfer 4.5 and 5.3 performed similarly, but for putaminal segmentation, FIRST was superior to Freesurfer 5.3.

The impact, however, of using different methods on the analyses of individual patients within a normative modeling framework is still unknown. This is relevant when volumetric MR data are used to generate normative distributions for both research and clinical use. In this study, we therefore aim to answer two research questions: 1) to what extent are methods interchangeable, as long as the same method is being used for deriving normative volume distributions and patient-specific volumes? and 2) can different methods be used for deriving normative volume distributions and patient-specific volumes? To answer these questions, we evaluated five state-of-the-art segmentation methods (Bron et al., 2014; Wenzel et al., 2018; Cardoso et al., 2015; Ledig et al., 2015; Fischl et al., 2002; Ikram et al., 2015).

## 2 Material and Methods

### 2.1 Data

To derive the normative distributions as a function of age, we applied the brain region segmentation methods to a subset of the population-based Rotterdam Scan Study, a prospective longitudinal study among community-dwelling subjects aged 45 years and older (Ikram et al., 2015). This subset is uniformly distributed over age and consists of 988 T1w MR brain images from non-demented (ND) (425 male, age = 68.1 ± 13.0 years). The total sample size of the Rotterdam Scan Study is larger: as of July 2015, a total of 12,174 brain MR scans have been obtained on the research scanner in over 5,800 individuals (Ikram et al., 2015). The 988 subjects form a subset with uniform age distribution (433 male, age = 68.3 ± 13.0 (mean ± std)). We adopted this dataset from Huizinga et al. (2018). All brain images were acquired on a single 1.5T MRI system (GE Healthcare, US). The T1w imaging protocol was a 3-dimensional fast radiofrequency spoiled gradient recalled acquisition with an inversion recovery pre-pulse sequence (Ikram et al., 2015). The images were reconstructed to a voxel size of $0.5\times 0.5\times 0.8\;{\text{mm}}^{3}$ , and the number of voxels in each dimension was 512 × 512 × 192.

In addition, we used the brain images of 42 (25 male, age = 81.9 ± 4.9 years) patients with AD at the time of the MRI scan from the same imaging study. Different MR acquisition protocols may lead to different image contrasts, and since most automated methods are—partly or entirely—driven by the contrast in the image; this may influence the segmentation results. To rule out possible differences of the segmentation due to the acquisition protocol, the methods were applied to the same images, all acquired with the same acquisition protocol (Ikram et al. (2015)).

### 2.2 Brain Segmentation Methods

We applied five previously proposed brain segmentation methods to the imaging data. The following five segmentation methods, explained in detail later, were evaluated:

1. Multi-atlas registration combined with tissue segmentation for cortical regions, developed at Erasmus MC (EMC), the Netherlands;
2. Freesurfer 5.1 (FS), developed at the Athinoula A. Martinos Center for Biomedical Imaging at Massachusetts General Hospital, United States of America;
3. Geodesic information flows (GIF), developed at University College London, United Kingdom;
4. Multi-atlas label propagation with expectation–maximization–based refinement (MALP-EM), developed at Imperial College London, United Kingdom; and
5. Model-based brain segmentation (MBS), developed at Philips Research Hamburg, Germany.

The regions segmented by each method are shown in Table 1. Later, a short description of each method is given.

**TABLE 1:** Characteristics of each method. The input format of each method is a 3D NIFTI file.

Method	References	Used reference data	Method of segmentation	# Regions	Region description
EMC	Bron et al. (2014)	Hammers et al. (2003), Gousias et al. (2008)	Multi-atlas segmentation with majority voting for label fusion	83	Subcortical regions, cortical regions, ventricles, corpus callosum, substantia nigra, lobes, brain stem, and cerebellum
FS	Fischl et al. (2002)	Fischl et al. (2002)	Multi-atlas segmentation with a Bayesian approach for label assignment	261	Subcortical regions, cortical regions, ventricles, lobes, optic chiasm, ventral diencephalon, lesions, vessels, corpus callosum, choroid plexus, brain stem, and cerebellum
GIF	Cardoso et al. (2015)	Petersen et al. (2010), Marcus et al. (2007) and Neuromorphometrics4	Multi-atlas segmentation with heat-kernel–weighted label fusion	144	Subcortical regions, cortical regions, ventricles, optic chiasm, ventral diencephalon, lesions, vessels, lobes, brain stem, and cerebellum
MALP-EM	Ledig et al. (2015)	Marcus et al. (2007) and Neuromorphometrics4	Multi-atlas segmentation with label refinement using prior information	138	Subcortical regions, cortical regions, ventricles, lobes, brain stem, and cerebellum
MBS	Wenzel et al. (2018)	Petersen et al. (2010), an Alzheimer‘s disease study at the Lahey Clinic, Burlington, MA	Model-based segmentation using a pretrained shape-constrained deformable surface model	56	Subcortical regions, ventricles, corpus callosum, fornix, septum pellucidum, lobes, brain stem, pons, and cerebellum

EMC is the method Erasmus MC by Bron et al. (2014), FS is the method FreeSurfer by Fischl et al. (2002), GIF is the method geodesic information flows by Cardoso et al. (2015), MALP-EM is the method multi-atlas label propagation with expectation–maximization–based refinement by Ledig et al. (2015), and MBS is the method model-based segmentation by Wenzel et al. (2018).

#### 2.2.1 EMC

This method combines multi-atlas registration and voxel-wise tissue segmentation for cortical regions, and hippocampus and amygdala. Probabilistic tissue segmentations are obtained on the image to be segmented using the unified tissue segmentation method (Ashburner and Friston, 2005) of SPM8 (Statistical Parametric Mapping, London, United Kingdom). Thirty labeled T1-weighted MR brain images are used as atlas images (Gousias et al., 2008; Hammers et al., 2003). The atlas images are registered to the subjects’ image using a rigid, affine, and non-rigid transformation model consecutively, and a mutual information-based similarity measure. The subjects’ images are corrected for inhomogeneities to improve registrations using the N3 algorithm (Tustison et al., 2010). Labels are fused using a majority voting algorithm (Heckemann et al., 2006). For the cortical regions, as well as hippocampus and amygdala, the label-map is combined with the tissue map such that the brain region volumes are determined on gray matter voxels only. For subcortical regions, the volumes are determined with a multi-atlas segmentation only as the probabilistic tissue segmentation for these regions is inaccurate. A more detailed description of this method can be found in Bron et al. (2014).

#### 2.2.2 FS

Freesurfer is widely used neuroimaging software developed by the Laboratory for Computational Neuroimaging at the Athinoula A. Martinos Center for Biomedical Imaging at Massachusetts General Hospital. It has many applications, but in this work, we use the brain region segmentation method described in Fischl et al. (2002). The method defines the problem of segmentation using a Bayesian approach in which the probability is estimated of a segmentation, given the observed image. First, the image is transformed into the atlas space with an affine transformation. Manually labeled atlas images provide the prior spatial information of the brain regions. The final segmentation is estimated by combining this spatial information with the intensity distribution of each brain region in the individual image. (For more detailed information about this method, we refer the reader to Fischl et al. (2002).) In our experiments, we used FS version 5.1. The user is able to use his own atlas, however, we used the atlas provided by FS. This method is publicly available^3^.

#### 2.2.3 GIF

This method is atlas-based and uses the geodesic path of a spatially variant graph to propagate the atlas labels (Cardoso et al., 2015). The atlas image database contains 130 T1-weighted MR brain images of cognitively normal participants from the Alzheimer’s Disease Neuroimaging Initiative (ADNI) study and 35 T1-weighted MR brain images from 30 young controls of the OASIS database (Marcus et al., 2007). The labeled images are made publicly available by Neuromorphometrics^4^ under academic subscription, as part of the MICCAI 2012 Grand Challenge on label fusion. First, each atlas image is registered to the individual image using a non-rigid transformation. A morphological distance of this image to each atlas image is estimated using the displacement field resulting from the image registration and the intensity similarity. The segmentation is estimated by fusing the labels of the morphologically closest atlas images. (For more details about this method, we refer the reader to Cardoso et al. (2015).) This method is publicly available^5^.

#### 2.2.4 MALP-EM

Like EMC, this method also combines multi-atlas registration and voxel-wise tissue segmentation. The atlas database of this method consists of 35 manually annotated T1-weighted MR brain images of 30 subjects of the OASIS database, which are also part of the atlas images of the GIF method (see Section 2.2.3). The atlas images of these 30 subjects are transformed to the space of the image that is to be segmented. These transformations are obtained via a non-rigid image registration approach (Heckemann et al., 2010). The subjects’ brains are extracted using the method proposed in Heckemann et al. (2015). The resulting 30 label images are fused, and a probabilistic map of each brain region is obtained. The labels are refined using expectation–maximization (EM) (Leemput et al., 1999), a brain tissue segmentation technique based on the image intensities. (More details can be found in Ledig et al. (2015).) In our experiments, we used MALP-EM version 1.2. This method is publicly available^6^.

#### 2.2.5 MBS

The MBS method is based on the model-based brain segmentation presented in Wenzel et al. (2018). The model is shape-constrained and represented by a triangulated mesh of fixed topology. Shape variations are modeled by principal component analysis of manually annotated meshes of a set of training images, resulting in a point distribution model (PDM) with a mean mesh and shape modes (Cootes et al., 1992). To segment a new image, the mean mesh is placed within the image by a generalized Hough transform compensating global translation and translation. Subsequently, the mean mesh is adapted by a global affine transformation and then region-specific affine transformations by adding weighted shape modes. The global and local affine transform parameters and the mode weights are estimated using a boundary detection based, for example, on the local intensity gradient and a penalization component regularizing the mesh shape, including the PDM. Finally, in a deformable deformation step, triangles can adapt individually, leading to a close match of the model surface with the image boundaries.

A database of 96 3T scans following the MP-RAGE acquisition protocol, split over three vendors (GE, Siemens, and Philips) served as training data. These scans have been randomly selected from the ADNI study ( n=87 ) and an Alzheimer’s disease study at the Lahey Clinic, Burlington, MA ( n=9 ). Ground truth delineations mostly followed structure definitions of the CMA guidelines,^7^ with two exceptions: (1) lateral thalamus borders follow image contrast, which may deviate from the CMA description, and (2) hippocampus annotations follow the EADC-ADNI harmonized protocol^8^ (Boccardi et al., 2015a; Boccardi et al., 2015b). The training data and procedure are extensively described in Wenzel et al. (2018).

### 2.3 Regions of Interest

**FIGURE 1:**
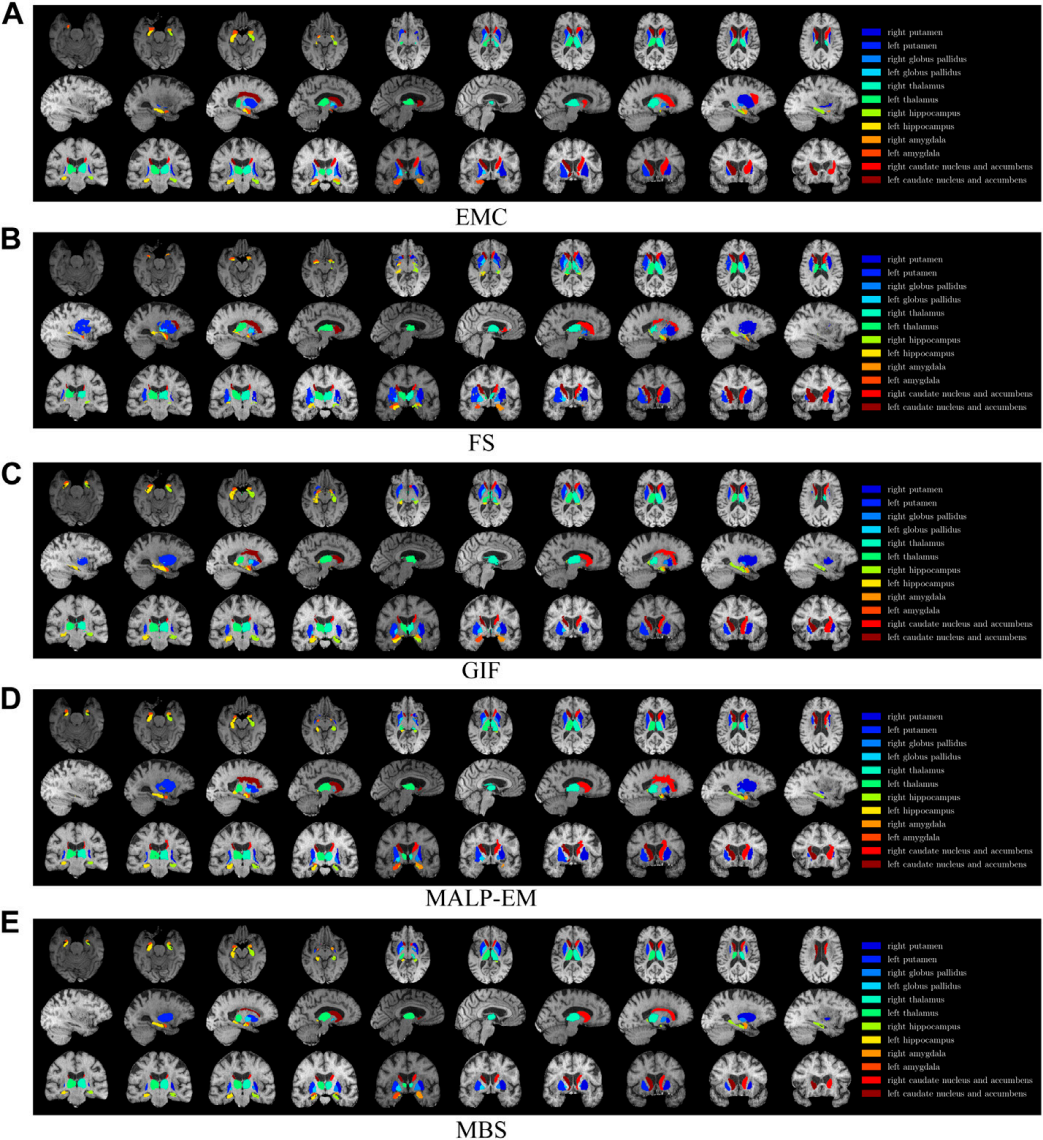
T1w MR brain image from one of the subjects, with a colored overlay of the brain regions analyzed in this work, segmented with all methods. Slices in the axial direction are shown in the top row, slices in the saggital direction are shown in the middle row, and slices in the coronal direction are shown in the bottom row. The legend on the right side shows the regions and their corresponding colors in the overlay. Note that only for this visualization, the segmentations were registered to the MNI space; some differences might be due to imperfections of this registration.

The set of brain regions in which each image is segmented differs per method. In this study, we focus on the following S=6 regions: hippocampus, amygdala, caudate nucleus and accumbens, putamen, thalamus, and globus pallidus. Figure 1 shows an example image of an ND subject with the analyzed brain regions in colored overlay. In the analysis, the volumes of the regions in the left hemisphere and the right hemisphere were summed. For all methods except MBS, the volume of the caudate nucleus was added to the accumbens volume because MBS already segments these as a single region.

### 2.4 Outlier Detection

Segmentation errors may occur due to bad image quality, pathology, or other method-related problems. These errors could lead to outliers in the volume data and may influence the statistics excessively. We therefore remove them from the volume data prior to the statistical analyses.

The segmentations of the ND subjects were not visually inspected as this would be too time-consuming. Method failures, that is, when the software pipeline did not result in a segmentation for the image, were excluded. On the remaining images, outliers were defined as having an absolute z-score higher than 5.0, derived with the population mean and standard deviation. Note that a z-score >5.0 does not necessarily imply a failed segmentation. We chose an absolute z-score of >5.0 , instead of the typical value of 3.0 because we wanted to include as much of the normal population as possible to generate the normative data, but we did not want to contaminate the normative data with unrealistic volumes. The segmentations of the AD patients were visually inspected, and obviously failed regions were excluded.

### 2.5 Statistical Analyses

In the analyses, two scenarios are considered: 1) both the normative volume distribution and the patient-specific volumes are calculated by the same method, and 2) the normative volume distribution and the patient-specific volumes are calculated by different methods. The requirements for two methods to yield comparable results under scenario 1) are given as follows:

i) a high correlation on the absolute volumes, measured with the Pearson’s correlation coefficient (PCC) and referred to as PCC-v;
ii) a high absolute agreement on the patient’s distances relative to the normative distribution, that is, a high absolute agreement on the patients’ z-scores, measured with the intraclass correlation coefficient (ICC) and referred to as ICC-z.

The requirements for two methods to yield comparable results under scenario 2) are given as follows:

i) a high absolute agreement on the absolute volumes, measured with the intraclass correlation coefficient (ICC) and referred to as ICC-v;
ii) a high absolute agreement on the patients’ z-scores, measured with the intraclass correlation coefficient (ICC) and referred to as ICC-z.

For scenario 2), requirement i naturally results in requirement ii. The requirements for scenario 2) are stricter than those for scenario 1). If in scenario 1), an offset or scaling is present in the volumes of different methods, the resulting patient’s z-score will be the same because the same method is used for comparing the patient to the normative distribution. However in scenario 2), absolute agreement on the volumes is necessary, that is, no offset or scaling is allowed for comparing the patient to the normative distribution as an offset or scaling will affect the patient’s z-score. The next sections describe how the normative distribution was established, how the correlation and absolute agreement are measured, and, in the case of scenario 1), how the diagnostic value of the z-scores was assessed.

#### 2.5.1 Normative Distribution Fitting

We fit an age-dependent normative distribution with the previously proposed LMS method (Cole and Green (1991)). This method assumes that the data are standard and normally distributed after applying the Yeo–Johnson transformation (Yeo and Johnson (2000)). The method estimates the λ− parameter of this transformation (L), the median (M), and coefficient of variation (S) for the appropriate volume at each age. With these three parameters, z-scores can be computed at each age. The smoothness of the resulting iso–z-score curves is influenced by the degrees of freedom δ, a user-defined parameter. In our experiments, we set the smoothness parameter δ to a value of 2. We used R-package VGAM for fitting these iso–z-score curves (Yee, 2010). The value of the brain region volume may also be influenced by other covariates than age, for example, gender and height. We correct for these covariates in the fitting procedure.

#### 2.5.2 Correlation and Absolute Agreement

To verify if scenario 1) is applicable, we first measure the correlation of the volumes calculated by the methods, with the Pearson’s correlation coefficient (PCC). We refer to these correlations as PCC-v. This coefficient is invariant for an offset and scaling of the data. To verify if scenario 2) is applicable, we compute the absolute agreement on the volumes, which was measured with the intraclass correlation coefficient (ICC). The type of ICC to be chosen depends on the problem at hand. McGraw and Wong (1996) give an overview of the possible ICCs. For the presented experiments, ICC(A,1) is the appropriate absolute agreement measure (McGraw and Wong, 1996). Let *X* be an n×k matrix where each column contains the measurements of a single method and each row contains the measurements of a single subject, then ICC(A,1) is given by McGraw and Wong (1996) is given as follows:

$$\text{ICC}\left(\text{A},1\right)=\frac{{\text{MS}}_{\text{R}}\left(X\right)-{\text{MS}}_{\text{E}}\left(X\right)}{{\text{MS}}_{\text{R}}\left(X\right)+\left(k-1\right){\text{MS}}_{\text{E}}\left(X\right)+\frac{k}{n}\left({\text{MS}}_{\text{C}}\left(X\right)-{\text{MS}}_{\text{E}}\left(X\right)\right)},$$ (1)

where ${\text{MS}}_{\text{R}}\left(X\right)$ is the mean square for rows, ${\text{MS}}_{\text{C}}\left(X\right)$ is the mean square for columns, and ${\text{MS}}_{\text{E}}\left(X\right)$ is the mean square error, which is defined as follows:

$${\text{MS}}_{\text{E}}\left(X\right)=\frac{1}{\left(n-1\right)\left(k-1\right)}\sum_{i,j=1}^{nk}{\left[X_{ij}-{\bar{X}}_{i}-{\bar{X}}_{j}+\bar{X}\right]}^{2},$$ (2)

where ${\bar{X}}_{i}=\frac{1}{k}\sum_{j=1}^{k}X_{ij}$ , ${\bar{X}}_{j}=\frac{1}{n}\sum_{i=1}^{n}X_{ij}$ , and $\bar{X}=\frac{1}{nk}\sum_{i,j=1}^{nk}X_{ij}$ . We refer to the absolute agreement on the volumes as ICC-v. The absolute agreement is maximal (1.0) when the measurements are exactly the same. When one or more measurements deviate, the absolute agreement is no longer 1.0 and drops according to how large the deviation is. A systematic error causing an offset in the measurements with a magnitude of, for example, the population standard deviation would lower the absolute agreement to ∼0.67. Or a scaling of the data by a factor of 1.2 would lower the absolute agreement to ∼0.7. The higher the ICC-v, the more reasonable it is to interchange methods.

We report all possible pairwise method combinations of PCC-v and ICC-v for M=5 methods for each of the *S* brain regions. Since the correlation and absolute agreement are determined with symmetric measures, we present PCC-v and ICC-v of the methods in a single 5×5 table, for each of the analyzed brain regions.

#### 2.5.3 Absolute Z-Score Agreement

To further assess the applicability of scenario 1), we also computed the absolute agreement on the AD patient z-scores with ICC(A,1). We indicated these values with ICC-z. We present ICC-z on AD subjects with PCC-v for ND subjects (see Section 2.5.2) in the same table, to facilitate their comparison.

### 2.6 AUC

To estimate how well the AD patient z-scores discriminate between normative volumes and patient-specific volumes in scenario 1), we determine the area under the receiver operating characteristic curve (AUC) of the z-score. The z-score was computed, as described in Section 2.5.1. The expected z-scores for the AD patients are <0, since we expect their brain structure volume to be lower than normal. We therefore define the AUC as the probability that a randomly chosen ND subject will have a higher z-score than a randomly chosen AD patient. The higher the AUC, the better will be the discrimination between AD patients and ND subjects. Since not every region is a known discriminative biomarker for AD, it is not necessarily expected that the AUC is high for each region. The hippocampus and amygdala are known to be discriminative biomarkers for AD, so for these regions, a high AUC is expected. For the computation of the AUC, only ND subjects within the age range of the AD patients, [71, 91] years, were included. A 95% confidence interval was computed by bootstrapping the z-scores 1,000 times.

## 3 Results

We used the following rating scale for PCC-v, ICC-v, and ICC-z, adopted from the rules of thumb in Mukaka (2012):

• Poor: <0.5

• Fair: 0.5−0.7

• Good: 0.7−0.9

• Excellent: >0.9

### 3.1 Outlier Detection

Method FS failed for nine ND subjects, either by not finishing the segmentation pipeline or by giving a zero volume output for some of the analyzed brain regions. Visual inspection of the MRI scans of these subjects did not show pathology or severe artifacts that would clearly explain failure. The method EMC failed for one ND subject, which was due to the failure of the brain extraction tool (Smith (2002)), which is used at the beginning of the pipeline. The remainder of the methods provided a segmentation for all images. The number of outliers per region and method on the remaining 978 subjects is reported in Table 2 Two T1w images of AD patients were excluded due to large scanning or motion artifacts. The number of failed segmentations per region and method in the remaining 40 images is shown in Table 3. In one image, there was a large lesion in the frontal lobe, affecting the segmentation of the caudate nucleus and accumbens of all methods. In one other image, the method MBS failed to segment the putamen and globus pallidus correctly.

**TABLE 2:** Number of outliers in the ND subjects per method for each brain region. The outliers were defined as having an absolute z-score >5.0 , derived with the population mean and standard deviation. The ten subjects that failed in the in the postprocessing were not included. As the outliers of the methods may overlap, the last column of the tables indicates the number of subjects included in the statistical analysis.

	EMC	FS	GIF	MALP-EM	MBS	TOTAL N
Hippocampus	0	0	0	0	0	978
Amygdala	0	1	1	0	0	976
Caudate nucleus and accumbens	2	1	0	2	0	975
Thalamus	0	1	0	0	0	977
Putamen	0	2	0	1	0	976
Globus pallidus	0	0	0	0	0	978

EMC is Erasmus MC by Bron et al. (2014), FS is FreeSurfer by Fischl et al. (2002), GIF is geodesic information flows by Cardoso et al. (2015), MALP-EM is multi-atlas label propagation with expectation–maximization–based refinement by Ledig et al. (2015), and MBS is model-based segmentation by Wenzel et al. (2018).

**TABLE 3:** Number of rejected segmentations in the AD subjects per method for each brain region, determined by visual inspection. The two subjects that failed in the postprocessing were not included. As the outliers of the methods may overlap, the last column of the tables indicates the number of subjects included in the statistical analysis.

	EMC	FS	GIF	MALP-EM	MBS	Total N
Hippocampus	0	0	0	0	0	40
Amygdala	0	0	0	0	0	40
Caudate nucleus and accumbens	1	1	1	1	1	39
Thalamus	0	0	0	0	0	40
Putamen	0	0	0	0	1	39
Globus pallidus	0	0	0	0	1	39

EMC is Erasmus MC by Bron et al. (2014), FS is FreeSurfer by Fischl et al. (2002), GIF is geodesic information flows by Cardoso et al. (2015), MALP-EM is multi-atlas label propagation with expectation–maximization–based refinement by Ledig et al. (2015), and MBS is model-based segmentation by Wenzel et al. (2018).

### 3.2 Volume Distributions

Table 4 shows the mean and standard deviation of the volumes of the ND subjects for each method and region. We performed a one-way ANOVA test, which showed that the p-values for each brain structure is p<0.05 , indicating that the volume distributions differ significantly between the methods. A multiple comparison post hoc analysis was done with the Tukey test. This test showed a limited number of non-significant differences, namely, the amygdala for methods EMC vs. GIF, the thalamus for methods FS vs. GIF and FS vs. MBS, and, finally, the putamen for methods FS vs. GIF. All other pairwise differences were statistically significant. The hippocampus volume of methods EMC and GIF deviates substantially from the other methods. The method EMC deviates due to a different definition of the hippocampus in the atlases that are used by the methods. The Hammers’ atlas (Hammers et al. (2003), Gousias et al. (2008)), used by the method EMC, defines the posterior border of the hippocampus such that the hippocampus tail is not included in the definition, whereas the other methods include the hippocampus tail. The method GIF deviates because it generally delineates the hippocampus in a larger volume. These same methods have a smaller average globus pallidus volume than the other methods. Visual inspection on a representative subset showed that these methods delineated a smaller globus pallidus. Methods MALP-EM and MBS calculated a smaller amygdala than the other methods.

**TABLE 4:** Mean (standard deviation) of brain region volumes in mm^3^ for the ND subjects.

	Hippocampus	Amygdala	Caudate nucleus and accumbens	Thalamus	Putamen	Globus pallidus
EMC	3,652 (494)	2,289 (320)	8,428 (1,265)	11,926 (1,637)	8,049 (1,139)	1897 (281)
FS	7,533 (1,166)	2,664 (402)	7,995 (1,154)	12,328 (1,614)	9,008 (1,338)	2,834 (480)
GIF	8,766 (906)	2,284 (269)	7,882 (1,059)	12,581 (1,333)	9,014 (1,090)	1735 (207)
MALP-EM	5,723 (862)	1887 (299)	7,640 (1,568)	13,678 (1,654)	7,427 (1,218)	2,472 (349)
MBS	6,052 (782)	1775 (243)	7,280 (895)	12,422 (1,451)	7,746 (977)	2,561 (304)

EMC is the method Erasmus MC by Bron et al. (2014), FS is the method FreeSurfer by Fischl et al. (2002), GIF is the method geodesic information flows by Cardoso et al. (2015), MALP-EM is the method multi-atlas label propagation with the expectation–maximization–based refinement by Ledig et al. (2015), and MBS is the method model-based segmentation by Wenzel et al. (2018).

**FIGURE 2:**
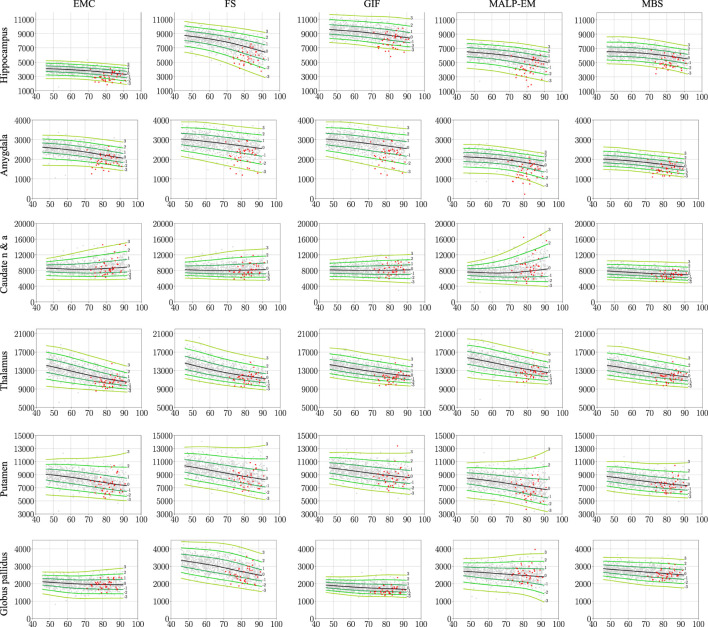
Normative brain structure volume distribution fitted on 978 ND subjects, visualized in iso-z-score lines from −3 to 3. All volumes are given in mm^3^ as a function of age [y]. The columns show volumes of each method, and the rows show the volumes per brain structure. The light gray scatters show the volumes of the ND subjects, and the red scatters show the volumes of the 40 AD patients, segmented with the same method as the normative distribution (scenario 1). The distribution was corrected for gender and height and is shown here for males of height 170 cm. EMC is the method Erasmus MC by Bron et al. (2014), FS is the method FreeSurfer by Fischl et al. (2002), GIF is the method geodesic information flows by Cardoso et al. (2015), MALP-EM is the method multi-atlas label propagation with expectation–maximization–based refinement by Ledig et al. (2015), and MBS is the method model-based segmentation by Wenzel et al. (2018). The caudate nucleus and accumbens was shortened to caudate n & a for visualization purposes.

Figure 2 shows the normative brain structure volume distribution fitted on 978 ND subjects, visualized in iso-z-score lines, for each method and brain structure. The red scatters show the volumes of the 40 AD patients, segmented with the same method as the normative distribution (scenario 1).

### 3.3 Correlation and Absolute Agreement

Table 5 present PCC-v and ICC-v for each pairwise combination of the five methods. For most regions, PCC-v was good ( ≥0.75 ) and was excellent for the region thalamus ( 0.91−0.97 ) and good to excellent for the putamen ( 0.88−0.96 ). For the three smallest structures, the hippocampus, amygdala and globus pallidus, ICC-v was generally poor, with some exceptions. The combination MALP-EM–MBS scored relatively high on ICC-v compared to the other method combinations. Visual inspection on a representative subset showed that the delineated hippocampus, amygdala, and globus pallidus for MALP-EM and MBS was similar in shape, explaining the good ICC-v. For the amygdala, the combination GIF–EMC also showed a good ICC-v. The three larger structures, the caudate nucleus and accumbens, thalamus, and putamen, showed generally higher ICC-vs. Visual inspection showed that their shape was, on average, more similar, possibly due to the less irregular shape of these regions than the smaller regions. Some method combinations showed poor ICC-v values for these larger regions, for example, MBS—EMC and MBS–MALP-EM for the caudate nucleus and accumbens, and GIF–MALP-EM for the putamen. MALP-EM–MBS also had a fair PCC-v for the regions caudate nucleus and accumbens; however, the other combinations showed a good PCC-v, indicating that the low ICC-v can mainly be explained by a volume offset and/or scaling.

**TABLE 5:** PCC-v (upper-right triangle) and ICC-v (lower-left triangle) of ND volumes.

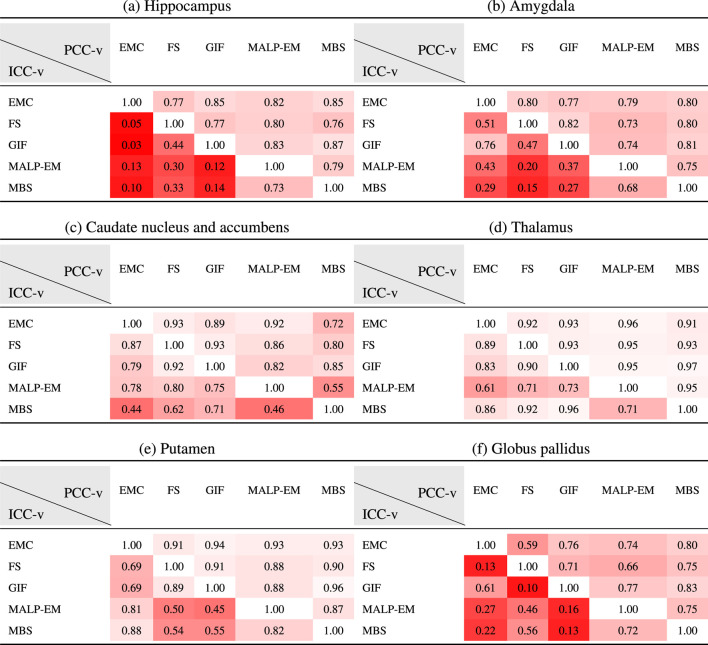

EMC is the method Erasmus MC by Bron et al. (2014), FS is the method FreeSurfer by Fischl et al. (2002), GIF is the method geodesic information flows by Cardoso et al. (2015), MALP-EM is the method multi-atlas label propagation with expectation–maximization–based refinement by Ledig et al. (2015), and MBS is the method model-based segmentation by Wenzel et al. (2018).

### 3.4 Absolute Z-Score Agreement

Table 6 shows ICC-z in the lower left triangle. In the upper-right triangle, PCC-v of the ND subjects is showed again, for easy comparison. ICC-z was good to excellent for regions thalamus ( 0.75−0.94 ) and putamen ( 0.83−0.96 ), fair to good for regions hippocampus ( 0.56−0.81 ), amygdala ( 0.65−0.88 ), and globus pallidus ( 0.50−0.72 ), and fair to excellent for the caudate nucleus and accumbens ( 0.51−0.96 ). The two method combinations with the lowest PCC-v of the caudate nucleus and accumbens, MBS–EMC and MBS–MALP-EM, also have the lowest ICC-z. This is also the case for the globus pallidus, where combinations EMC–FS and MALP-EM–FS have the lowest PCC-v and the lowest ICC-v.

**TABLE 6:** PCC-v of the ND volumes (upper-right triangle) and ICC-z of AD volume z-scores (lower-left triangle). The ICC-z is computed according to scenario 1.

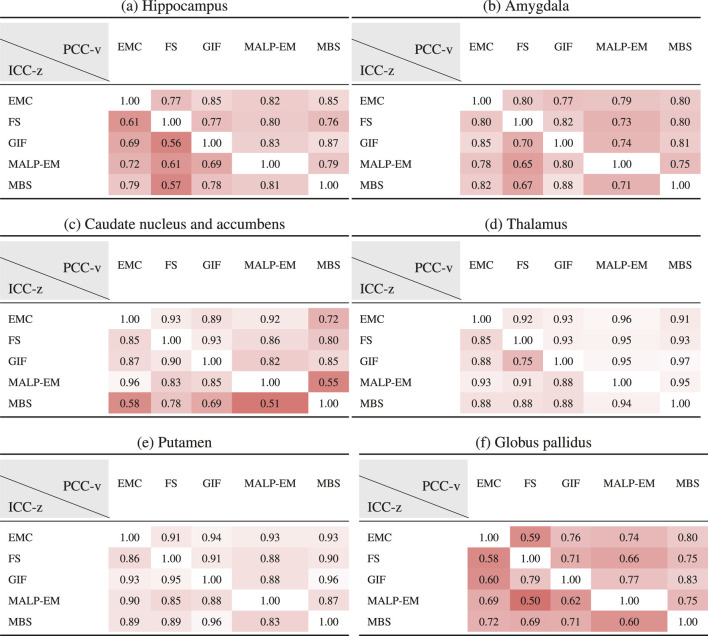

EMC is the method Erasmus MC by Bron et al. (2014), FS is the method FreeSurfer by Fischl et al. (2002), GIF is the method geodesic information flows by Cardoso et al. (2015), MALP-EM is the method multi-atlas label propagation with expectation–maximization–based refinement by Ledig et al. (2015), and MBS is the method model-based segmentation by Wenzel et al. (2018).

### 3.5 AUC

Table 7 shows the AUC for each method and brain region. The highest AUC was achieved for the hippocampus (on average 0.79) and amygdala (on average 0.78), demonstrating their involvement in AD. For the thalamus and putamen, the AUC was >0.5 for all methods, indicating that these regions are also affected by AD. For the method GIF, the AUC of regions thalamus and globus pallidus were high compared to the other methods. The methods FS, MBS, and GIF had comparable thalamus volumes for the ND subjects, but the AD thalamus volumes segmented by GIF were, on average, 120 mm^3^ lower than those segmented by MBS and 50 mm^3^ lower than those segmented by FS. The methods EMC and GIF had comparable globus pallidus volumes for the ND subjects, but for AD subjects, the volumes segmented by GIF were, on average, 320 mm^3^ lower than those segmented by EMC.

**TABLE 7:** AUC (95% confidence interval) for all regions, where the volumes of the normative distribution and the AD patients were generated by the same method (scenario 1).

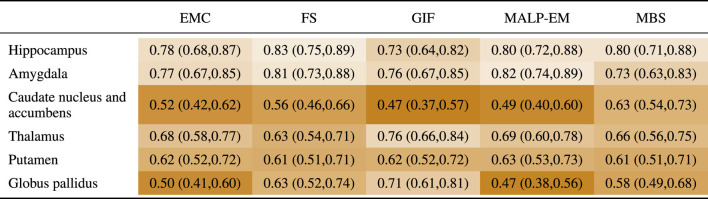

EMC is the method Erasmus MC by Bron et al. (2014), FS is the method FreeSurfer by Fischl et al. (2002), GIF is the method geodesic information flows by Cardoso et al. (2015), MALP-EM is the method multi-atlas label propagation with expectation–maximization–based refinement by Ledig et al. (2015), and MBS is the method model-based segmentation by Wenzel et al. (2018).

### 3.6 Computational Efficiency

All methods were executed on a Linux Sun Grid Engine (SGE) computing cluster with eight computing nodes, each having multiple cores. All methods, except FS, provide an option for using multiple cores. This is especially efficient for methods that use multi-atlas registration, where the registrations of the subjects in the atlas database can run in parallel. In practice, the method GIF had the longest computation time, despite the usage of multiple cores. This was mainly due to the non-rigid image registrations of the 165 images in the atlas database. The method MBS was most efficient, needing only a few minutes to segment all 56 regions in a brain image on a single core. Except for MALP-EM, needing 33 GB of RAM per brain image, the memory usage of the methods was modest (≤8 GB) for the hardware in modern computers.

## 4 Discussion

We evaluated the correlation and absolute agreement on regional volumes computed with different automated brain segmentation methods, and the impact of the volume differences between these methods on single-subject analysis in a normative modeling framework. We evaluated two scenarios: 1) The normative volume distributions and the patient-specific volumes were calculated by the same method, and 2) the normative volume distributions was calculated by a different method than the patient-specific volumes. To this end, we applied five state-of-the-art automated brain segmentation methods on the T1w MR brain images of 988 ND subjects, and 42 AD patients acquired with the same MR acquisition protocol.

The PCC-v showed that the volumes of all regions correlated well, indicating that volume differences between methods in ND subjects are mainly due to systematic differences, such as the usage of different atlases and region definitions. The ICC-v however was generally low, especially for the smaller regions, including the hippocampus, amygdala, and globus pallidus. The low ICC-v indicates that the methods cannot be interchanged in a normative modeling framework and scenario 2) is not applicable. This also becomes visually clear from Figure 2, when comparing the location of the red dots across graphs in a row.

The ICC-z, with which the agreement on the AD patient position relative to the normative distribution was measured in the case of scenario 1), was good to excellent for the thalamus and putamen, which also showed a good to excellent PCC-v. The other four regions showed lower ICC-z, indicating that different methods would result in different AD patient positions relative to the normative distribution, even when the normative distribution was computed using the same method as the patient data. A low PCC-v also seemed to result in a low ICC-z. A high PCC-v however does not necessarily result in a high ICC-z. This may indicate that brain morphology changes because AD affects each method differently.

The AUC, with which the z-score discrimination between the patient and normative volumes was measured in the case of scenario 1), was relatively high for the regions hippocampus and amygdala for all methods, demonstrating the involvement of these regions in AD. For the method GIF, the thalamus volume showed to be a better discriminator for AD than the hippocampus volume, which is unexpected, as this region is not known for its involvement in AD, and the other methods did not show such a high AUC for the thalamus. A possible explanation is that the method GIF is more affected than the other methods by the brain morphology change due to AD, such as larger ventricles.

Several limitations of this study can be highlighted. First, the segmented results rely strongly on the atlas that was used by the method. As was shown with the hippocampus, differences in volume may be largely explained by the atlas and how the region was defined. For this reason, operationalized and quantitated landmark differences to help a Delphi panel converge on a set of landmarks on the hippocampus and provided a set of manually segmented images for training models for automatic hippocampus segmentation. In this study however, we considered the atlas a part of the method, and we did not study specific atlas-related volume differences. Second, the number of AD patients was limited, which limits the generalization of the conclusions drawn from these results. In future studies, a higher number of AD patients should be used to generalize the study results. Third, we used images that were acquired on a single 1.5 T scanner with the same acquisition protocol. This allowed us to study the effect of differences in segmentation methods, while not considering the confounding effect of differences in acquisition protocols. Future research should investigate how differences in acquisition protocols influence the comparison of individual patients to normative data and to study the generalizability of our results in more heterogeneous datasets. Previously, tools have been developed to cope with volumetric differences due to scanning artifacts. The effectiveness of these tools can be tested using our research setup with normative data. Finally, we limited our study to five automatic segmentation methods. Many more have been previously proposed, and it remains an active area of research, particularly since the rise of deep learning techniques (Bao and Chung, 2018; Shakeri et al., 2016). These methods may achieve higher accuracy and precision, and therefore, the AUC of the AD patient z-scores may increase. Future studies should therefore also include deep learning–based approaches.

### 4.1 Conclusion

In this study, we aimed to answer two research questions: 1) to what extent are methods interchangeable, as long as the same method is being used for computing normative volume distributions and patient-specific volumes? and 2) can different methods be used for generating normative volume distributions and patient-specific volumes? Based on the absolute agreement results on the volume data of 988 non-demented subjects, we conclude that it is essential that the same method is used to generate normative volume distributions and patient-specific volumes. For most regions, the correlation was good ( >0.75 ), indicating that volume differences between methods in ND subjects are mainly due to systematic differences. When the same method is being used for generating normative and patient data, we found that the agreement on the AD patient’s position relative to the normative distribution (ICC-z) was high for the regions thalamus and putamen. Our results are encouraging as they indicate that the studied methods are interchangeable for these regions. For the regions hippocampus, amygdala, caudate nucleus and accumbens, and globus pallidus, not all method combinations showed a high ICC-z. Whether two methods are indeed interchangeable should be confirmed for the specific application and dataset of interest.

## Data Availability

The datasets presented in this article are not readily available because of restrictions based on privacy regulations and informed consent of the participants. Requests should be directed toward the management team of the Rotterdam Study (secretariat.epi@erasmusmc.nl), which has a protocol for approving data requests.

## Footnotes

1
http://dbm.neuro.uni-jena.de/wordpress/vbm/

2
https://fsl.fmrib.ox.ac.uk/fsl/fslwiki

3
http://freesurfer.net/

4
http://neuromorphometrics.com/

5
http://cmicti g.cs.ucl.ac.uk/niftyweb/program.php?p=GIF

6
https://github.com/ledigchr/MALPEM

7
https://web.archive.org/web/20180226014735/http://www.cma.mgh.harvard.edu/manuals/

8
http://www.hippocampal-protocol.net/SOPs/index.php
